# Influence of electrospray deposition on C_60_ molecular assemblies

**DOI:** 10.3762/bjnano.12.45

**Published:** 2021-06-15

**Authors:** Antoine Hinaut, Sebastian Scherb, Sara Freund, Zhao Liu, Thilo Glatzel, Ernst Meyer

**Affiliations:** 1 Department of Physics, University of Basel, Klingelbergstrasse 82, 4056 Basel, Switzerland

**Keywords:** alkali halide, Au(111), bulk insulator, C_60_, electrospray, electrospray deposition, fullerene, high-vacuum electrospray deposition (HV-ESD), molecular assembly, nc-AFM, NiO, single molecule, thermal evaporation

## Abstract

Maintaining clean conditions for samples during all steps of preparation and investigation is important for scanning probe studies at the atomic or molecular level. For large or fragile organic molecules, where sublimation cannot be used, high-vacuum electrospray deposition is a good alternative. However, because this method requires the introduction into vacuum of the molecules from solution, clean conditions are more difficult to be maintained. Additionally, because the presence of solvent on the surface cannot be fully eliminated, one has to take care of its possible influence. Here, we compare the high-vacuum electrospray deposition method to thermal evaporation for the preparation of C_60_ on different surfaces and compare, for sub-monolayer coverages, the influence of the deposition method on the formation of molecular assemblies. Whereas the island location is the main difference for metal surfaces, we observe for alkali halide and metal oxide substrates that the high-vacuum electrospray method can yield single isolated molecules accompanied by surface modifications.

## Introduction

Electrospray deposition in high vacuum (HV-ESD) is a well-established technique for the introduction of molecules into high-vacuum environments and the deposition of these molecules on surfaces [1–3]. Based on electrospray ionisation [4], HV-ESD gives the possibility to study complex or fragile molecules that are impossible to safely deposit onto surfaces with traditional deposition techniques. So far, using HV-ESD, numerous molecular species with potential applications in biology and photovoltaics, or with magnetic or thermal expansion properties have been deposited on a variety of materials, ranging from metal surfaces [5–13], over metal oxides [14] and insulating substrates [15] to graphene monolayers on metals [16].

**Figure 1:**
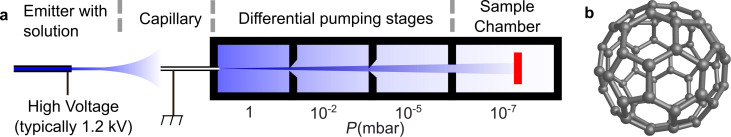
(a) Scheme of the high-vacuum electrospray deposition device. Typical working pressures of the different chambers are indicated. (b) Representation of a C_60_ molecule.

In HV-ESD-based devices, a solution containing the molecules reaches an emitter located in front of the entrance capillary, as shown in Figure 1a. Then, by applying a voltage difference, typically 1.2 kV, between the solution and the capillary, droplets of solvent and diluted molecules are created and accelerated towards the capillary, through the differential pumping vacuum system, finally reaching the sample in ultrahigh vacuum. The main requirement for the suitability of molecules for ESD is their solubility in a compatible solvent. A drawback is, therefore, the presence of the solvent itself. Various implementations of electrospray deposition setups were developed to allow for the selection of the molecular species via mass spectrometer filtering, showing successful depositions [8,17–18]. When no filtering is added, the setup is working in an all-in-line configuration as shown in Figure 1a. In such a situation, all species introduced in the vacuum that are not evacuated via the pumping system or adsorbed to a wall of the device can reach the sample. Nevertheless, the contamination from solvent introduction can be reduced down to conditions compatible with high-resolution scanning probe microscopy (SPM) techniques [10,12].

Buckminsterfullerene C_60_, scheme in Figure 1b, is among the most extensively studied molecules in surface science, especially in SPM under UHV conditions. The ease of its thermal evaporation, the organised structure generally obtained, and the potential of its uses have made C_60_ a model case for on-surface molecular studies [19–27]. Two-dimensional C_60_ layers have been observed on metals [20,25,28] and metal oxide semiconductors [23–24], while large three-dimensional molecular islands or clusters have been revealed on ionic crystals or bulk insulators [22,26–27,29]. Most of the studies have been performed after thermal evaporation (TE) of C_60_ from a crucible, but C_60_ is also one of the first molecules studied in HV-ESD experiments [5,30].

Here, we present a comparison between TE and HV-ESD regarding the adsorption and structure formation of C_60_ molecules on surfaces at low coverages, that is, below one monolayer down to single molecules. We used a non-contact atomic force microscope (nc-AFM) working at room temperature to study formation and shape of C_60_ islands on three substrates with different intrinsic properties. These are, first, Au(111), a metal surface widely used in SPM studies, second, KBr(001), a bulk insulator allowing for the decoupling of molecular species and used as model surface in nc-AFM measurements [31–34], and, finally, NiO(001), a p-type wide-bandgap metal oxide with potential applications in photovoltaics [35–37]. For all cases, we show the typical C_60_ structures formed by TE and compare these with the results from HV-ESD. This allows us to discuss the influence of the HV-ESD method for the different surfaces.

## Results and Discussion

### C_60_ on Au(111)

**Figure 2:**
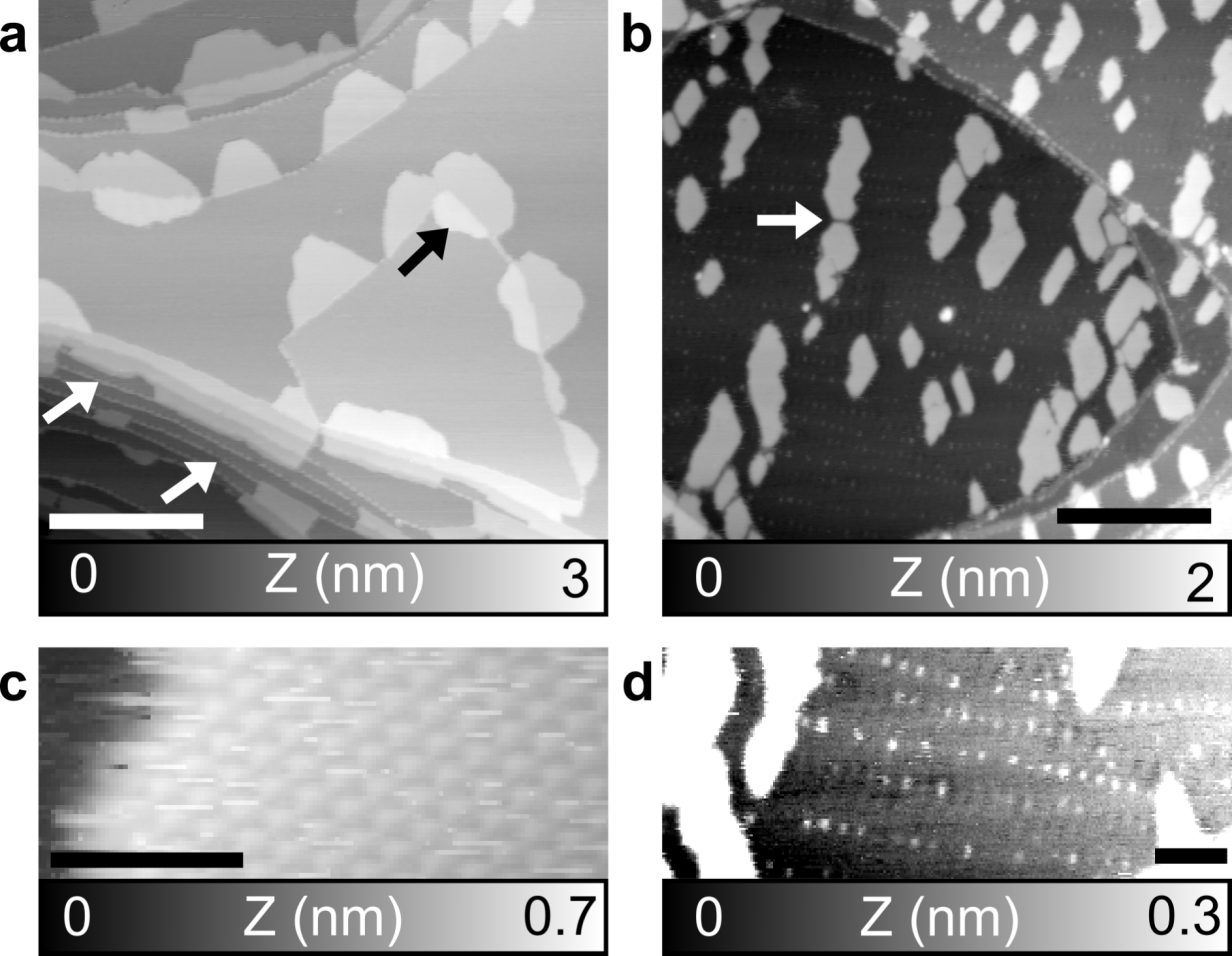
C_60_ on a Au(111) surface. (a) After TE and (b) After HV-ESD. (c) Zoom on an island after HV-ESD. (d) Zoom on a Au(111) terrace with covered herringbone kinks, after HV-ESD. The contrast has been highly modified. Parameters: (a) *f*_2_ = 0.961 MHz, *A*_2_ = 600 pm, Δ*f*_2_ = −40 Hz; (b,d) *f*_1_ = 153 kHz, *A*_1_ = 5 nm, Δ*f*_1_ = −15 Hz; (c) *f*_2_ = 1.079 MHz, *A*_2_ = 500 pm, Δ*f*_2_ = −60 Hz. Scale bar: (a, b) 100 nm, (c) 5 nm, and (d) 25 nm.

The deposition of C_60_ molecules on a Au(111) surface at room temperature via TE is known to lead to the formation of monolayer islands until the surface is fully covered [21,25,28]. A nc-AFM topography image of a Au(111) surface covered with 0.35 monolayers of C_60_ molecules is shown in Figure 2a. Large clean terraces separated by monoatomic step edges are observed. On top, the adsorbed C_60_ molecules are seen in two possible locations. First, all step edges of the surface are filled with C_60_ (see white arrows). Step edges are known to be favorable anchoring sites and to easily trap molecules. Second, C_60_ molecules are observed in islands formed on the Au(111) surface and aligned along the step edges of the surface. Islands are observed at the bottom or on top of step edges with few of them on both sides (see black arrow). These islands, similar to what has been already reported in literature are monolayers, the size of which depends on the coverage [21,25,28]. In our case, with a coverage of 0.35 monolayers, their average size is 1500 nm^2^.

At high coverages, C_60_ molecules deposited on Au(111) surfaces with HV-ESD are known to form large assemblies [5]. A Au(111) surface with a coverage of 0.30 monolayers of C_60_ after HV-ESD, similar to that after TE in Figure 2a, is shown in the topography map in Figure 2b. Monoatomic step edges and terraces of a few hundreds of nanometers in size are observed, suggesting a limited influence of the HV-ESD method. The C_60_ molecules cover step edges and form monolayer islands, similar to TE. High-resolution imaging of the islands, shown in Figure 2c, confirms the hexagonal lattice arrangement of C_60_ with a lattice parameter close to 1 nm. An important difference is that many of the C_60_ islands are observed in the middle of the terraces, that is, far away from step edges. This phenomenon, not observed for TE, suggests a difference in the nucleation during island formation. It could be explained by the presence of defects on the surface. These defects, induced by the HV-ESD method itself, could allow for the trapping of C_60_ molecules and island nucleation far away from step edges. The separation distance between islands can also be small, as indicated by the white arrow and observed in several place in Figure 2b. Another difference compared to TE is the size and numbers of the islands. At a coverage of 0.30 monolayers, the average size of the islands after HV-ESD is 600 nm^2^, that is, by a factor of 2.5 smaller than after TE. This reduced size also indicates the presence of additional nucleation sites that can facilitate the formation and stabilization of these smaller islands.

At last, one has to mention the effect of the HV-ESD method on surface pollution. This is revealed by some small dots, forming lines as shown on the Figure 2d extracted from Figure 2b but with an enhanced contrast. Theses dots are located on the elbows of the herringbone reconstruction, a favorable trapping site [38]. The height of the dots is too small to be C_60_ molecules and the dots are therefore attributed to solvent. Nevertheless, such defects can influence the nucleation and the size of C_60_ islands. The presence of the solvent on the surface can also be increased, as shown in part 1 of Supporting Information File 1, but shape, size, and distribution of the C_60_ islands are still preserved.

We have shown the limited influence of the HV-ESD method on the C_60_ island structures. Interestingly, a reduced size and a more dispersed distribution of the islands is found. Similar results are obtained for a Ag(111) surface, as shown in part 2 of Supporting Information File 1. The absence of favorable anchoring sites, similar to kinks in the herringbone reconstruction, on Ag(111) suggests that not only the adsorbed solvent molecules are responsible for the nucleation of islands in the middle of the terraces.

### C_60_ on KBr(001) surface

**Figure 3:**
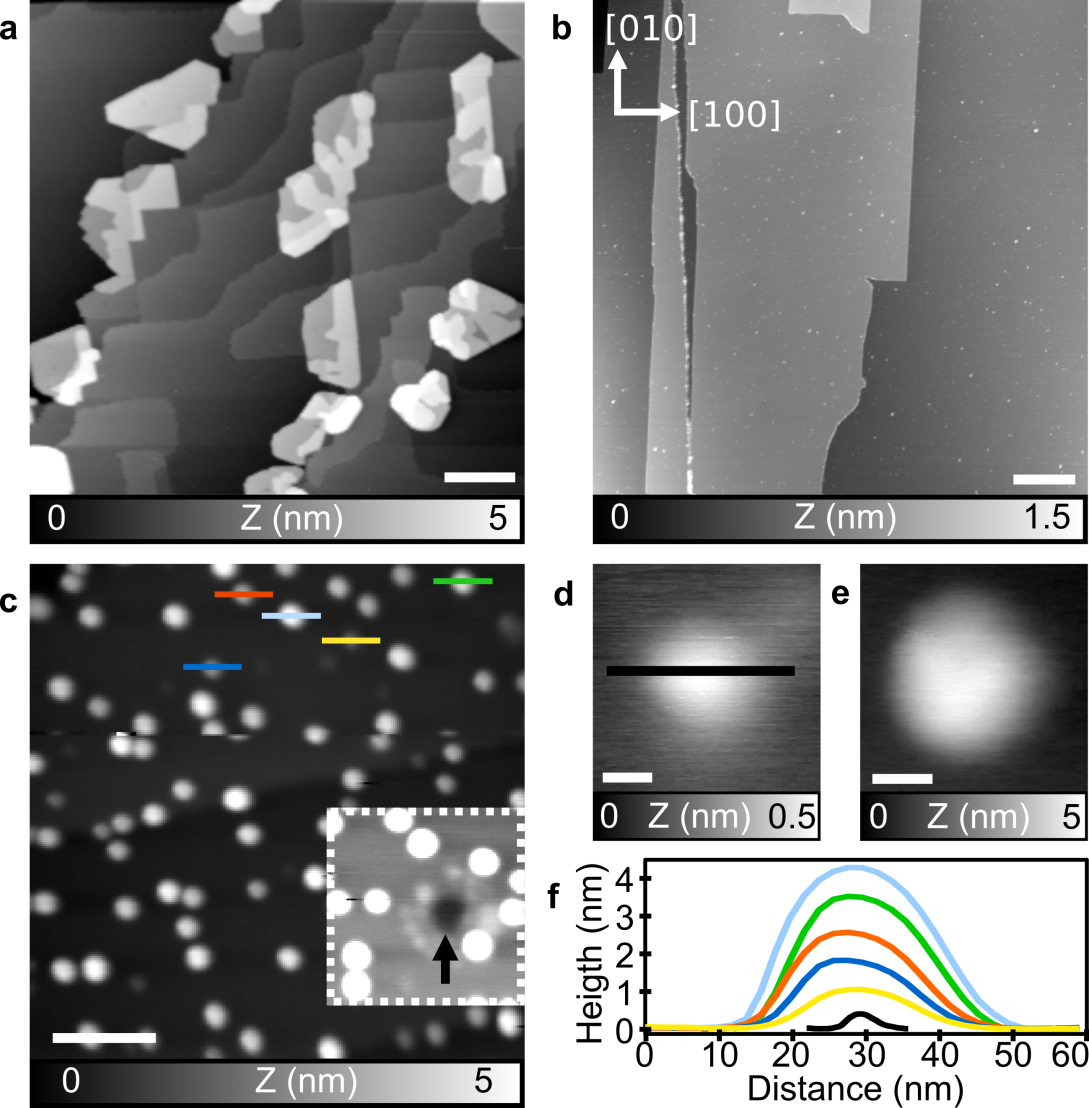
C_60_ on a KBr(001) surface. (a) After TE, (b) after low-coverage HV-ESD, and (c) after high-coverage HV-ESD; inset: area with a modified contrast. (d) Zoom on a small cluster from (b). (e) Zoom on an island from (c). (f) Corresponding height profiles from (c) and (d). Parameters: (a) *f*_1_ = 156 kHz, *A*_1_ = 4 nm, Δ*f*_1_ = −5 Hz; (b, d) *f*_2_ = 1.079 MHz, *A*_2_ = 800 pm, Δ*f*_2_ = −30 Hz; (c, e) *f*_1_ = 152 kHz, *A*_1_ = 8 nm, Δ*f*_1_ = −8 Hz. The crystal lattice orientation is shown in (b). Scale bar: (a–c) 100 nm, (d) 2 nm, (e) 10 nm.

The deposition of C_60_ on bulk insulators is known to lead to the creation of large islands [22,31]. A typical KBr(001) surface after TE of C_60_ is shown in the nc-AFM topography image in Figure 3a. Large clean terraces separated by monoatomic steps edges are observed. The preferential alignment of the step edges is along the [110] directions. C_60_ islands are found distributed on all surfaces, but always along a step edge. They present favorable edge directions, following the sixfold symmetry of the C_60_ lattice. The height of the C_60_ islands is about three monolayers. A dewetting process is also observed [22,26] as visible by the more round shape of the second- and third-layer step edges of C_60_ islands.

The deposition of C_60_ on KBr(001) via HV-ESD is more challenging. The insulating nature of the KBr substrate can lead to a charging of the surface induced by the deposition of charged species. This can have a strong influence on the surface local charge and, eventually, on island formation [15] during HV-ESD. To improve scan conditions, imaging was performed a few hours after HV-ESD to reduce the charging effects [39].

A low coverage of C_60_ on KBr(001) obtained after HV-ESD is shown in the topography image of Figure 3b. The KBr(001) surface presents similar large terraces and step edges as clean KBr(001). The C_60_ molecules are forming small cluster visible as many small dots dispersed on the terraces or along step edges. A zoom on such a cluster is shown in Figure 3d. The presence of these small clusters indicates a low diffusion of the C_60_ molecules, contrary to the TE deposition.

At higher coverages, but still below one monolayer, C_60_ molecules form islands dispersed on the surface, as shown in Figure 3c. The islands are clearly different to what is obtained after TE of C_60_, also shown by the zoom on such an island in Figure 3e. Their average contact area, that is, the surface occupied by the first layer on the KBr surface, is about 1100 nm^2^, less than a tenth of what is obtained after TE (12000 nm^2^). Their height distribution is enlarged compared to islands formed after TE, suggesting a growth mechanism that favors a 3D growth over layer-by-layer growth. A few profiles acquired on C_60_ islands in Figure 3c are shown in Figure 3f and compared to the profile acquired on the small cluster of Figure 3d. The number of layers ranges from one to at least five. Moreover, the islands present a more rounded shape, compared to TE islands, and no direction favored by the sixfold symmetry is observed.

The influence of HV-ESD on the surface itself can also be seen. First, monolayer-deep pits are visible, see the arrow in the contrast-modified inset of Figure 3c and in part 3 of Supporting Information File 1. They are similar to the pits created after electron and ion bombardment [40–43] or low-temperature plasma exposure of such a surface [44]. Such defects are known to increase molecular trapping and their creation is therefore studied for those reasons [32,43,45–46]. In HV-ESD deposition, their presence can be reduced but not inhibited without annealing of the surface [44].

### C_60_ on NiO(001) surface

NiO is a wide-bandgap metal oxide with potential applications in organic photovoltaics [47]. To date, only few SPM studies have focused on the adsorption of organic molecules on NiO surfaces [35–37]. Because organic dyes are large and complex molecules, their TE is impossible, making HV-ESD methods the only deposition technique compatible with fundamental studies. A first step is the study of the HV-ESD influence on the deposition of a simple well-known molecule.

**Figure 4:**
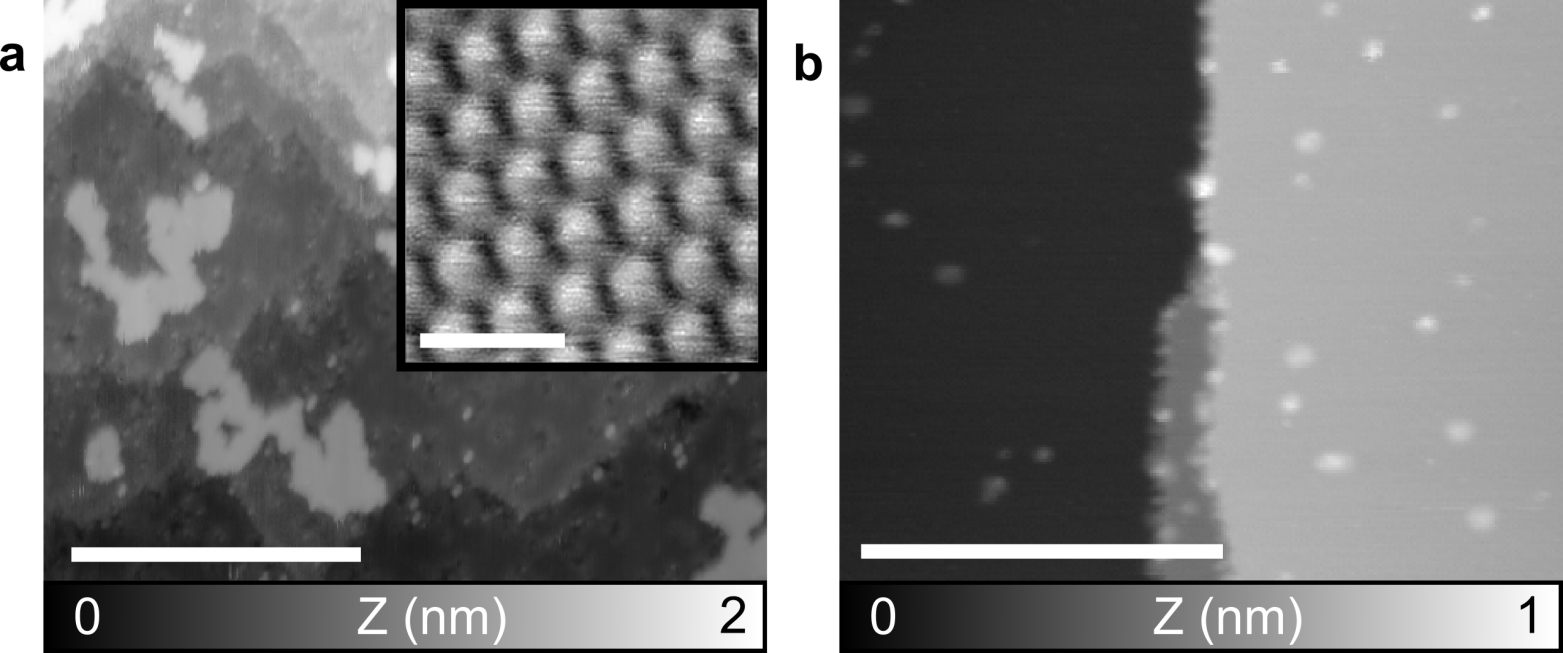
C_60_ on a NiO(001) surface. (a) Large scale topography nc-AFM image after TE; inset: zoom on an island with molecular resolution. (b) Topography nc-AFM image after HV-ESD. Parameters: (a) *f*_1_ = 169 kHz, *A*_1_ = 8 nm, Δ*f*_1_ = −5 Hz; (b) *f*_2_ = 1.057 MHz, *A*_2_ = 800 pm, Δ*f*_2_ = −10 Hz. Scale bar: (a, b) 50 nm, inset 2 nm.

A nc-AFM topography image of a NiO(001) surface after the deposition of C_60_ via TE is shown in Figure 4a. Terraces in the NiO(001) surface are observed, separated by monoatomic steps. Also, because of the high reactivity of the surface, the presence of defects, already reported [37] and visible as small holes, should be noted. C_60_ on the surface can easily be identified as large bright areas corresponding to monolayer islands. These islands show irregular contour paths without preferential orientation as well as holes free of molecules. A zoom on such an island is displayed in the inset. The typical hexagonal lattice with a distance of 1 nm between C_60_ molecules is observed. The islands are also distributed on the whole surface and their area depends on the size of the terrace they lie on, that is, the island size increases with the terrace size. This observation is compatible with the diffusion of molecules on terraces but not over step edges, yielding a larger number of molecules and, therefore, larger islands on larger terraces. Finally, small protrusions are often observed close to defects possibly corresponding to small clusters of molecules.

The possibility to perform HV-ESD on a NiO surface is of interest for the elaboration of p-type solar cell devices [36–37,48]. The nc-AFM topography image of Figure 4b is obtained after HV-ESD of C_60_ on a clean NiO(001) surface. Even though the high-quality cleavage of the surface, visible by the large terrace, would enable C_60_ molecules to form large islands via diffusion, no islands are observed. Only small protrusions can be distinguished. The small size of these protrusions is compatible with small clusters or single molecules.

The influence of the ESD is limited for the NiO(001) surface. No major impact, similar to KBr, is observed. Also, neither a solvent layer nor the charging of the sample because of deposited ions are observed. Therefore, the observation of single C_60_ molecules on the NiO(001) surface is the major outcome of HV-ESD. The presence of single molecules at room temperature hints at a reduced diffusion. A possible explanation is the creation of defects during HV-ESD favoring the trapping of molecules directly upon landing.

## Conclusion

We show the influence of HV-ESD on the surface preparation of molecular layers. This method is known to be a good alternative to TE when working with non-volatile molecules and has proven to be compatible with sensitive techniques, such as low-temperature AFM with CO tip imaging [10,12]. Nevertheless, we have shown some influence on the surfaces and the formation of molecular assemblies that should not be neglected when studying molecular structures, especially, when no post-deposition treatment is carried out. The influence of HV-ESD on the surfaces themselves is negligible under proper conditions. The influence on the molecular assemblies depends on the surface. For metals, HV-ESD is found to reduce the size of the islands. For the alkali halide KBr and the metal oxide NiO, the structure size is reduced down to single molecules. In all cases, the creation of defects, possibly combined with remaining solvent, reduces the diffusion length of the molecules. For studies in which large molecular structures are needed, a gentle annealing eliminates the spray influence. Additionally, when focusing on single molecules, small aggregates, or islands, the HV-ESD method is well suited and could open new possibilities to stabilize single molecules at room temperature.

## Experimental

### Sample preparation

Au(111) single crystals (Mateck GmbH) were prepared under UHV conditions by several cycles of Ar^+^ sputtering and annealing at 750 K. KBr(001) crystals (Mateck GmbH) were prepared either by cleavage in air and quick introduction in UHV or by cleavage under UHV conditions. Subsequently, annealing at 350 K for 2 h was carried out. NiO(001) crystals (Surfacenet) were prepared by annealing at 870 K until a low pressure (10^−9^ mbar) was reached, followed by the cleavage under UHV conditions and a second annealing at 770 K for 1 h. In all cases, atomically flat surfaces with large terraces separated by atomic steps were obtained.

### Room-temperature AFM

Room-temperature nc-AFM measurements were performed with a custom-built non-contact atomic force microscope with Nanonis electronics RC5. PPP-NCL cantilevers (Nanosensor) were used as sensor (typical resonance frequency of *f*_1_ = 170 kHz, oscillation amplitude *A*_1_ = 2–5 nm, and *f*_2_ = 1 MHz, *A*_2_ = 400–800 pm. Their preparation consisted of annealing for 1 h at 400 K followed by tip Ar^+^ sputtering for 90 s at 680 eV at an Ar^+^ pressure of 3 × 10^−6^ mbar. The base pressure of the UHV system during AFM measurements is maintained at 2 × 10^−11^ mbar.

### Electrospray deposition

Electrospray deposition was performed on samples kept at room temperature using a commercial system from MolecularSpray [49]. The setup, shown in Figure 1a, is connected to the preparation chamber of the system. It is based on a straight-line succession of three chambers as represented in Figure 1b. When connected, the vacuum level of the sample chamber is 1 × 10^−7^ mbar. The C_60_ molecules were dissolved in a toluene/methanol mixture (ratio 5:1 in volume). During spray deposition the pressure rose up to 1 × 10^−6^ mbar. The typically applied voltage was 1.2 kV with occasional necessary adjustments during spray deposition to maintain stable conditions. Deposition times were typically around 10 min.

### Thermal evaporation

C_60_ was evaporated from a quartz crucible in a Kentax evaporator onto samples kept at room temperature. Evaporation was calibrated using a quartz microbalance and was performed at 410 °C for 3 min.

## Supporting Information

Supporting Information features additional images of the influence of HV-ESD on surfaces and assembly formation. Part 1 describes the Au(111) surface with a significant presence of solvent. Part 2 presents a comparison between HV-ESD and TE for the Ag(111) surface. Part 3 shows defect formation after HV-ESD on a KBr surface.

File 1 Additional experimental data
