# Supplementary material for: Influence of electrospray deposition on C60 molecular assemblies
Source: Beilstein J Nanotechnol. 2021 Jun 15;12:552–8. doi: 10.3762/bjnano.12.45 (PMC8218541; doi:10.3762/bjnano.12.45)
Supplement: File 1 — Additional experimental data [file Beilstein_J_Nanotechnol-12-552-s001.pdf]

BEILSTEIN JOURNAL OF NANOTECHNOLOGY

Supporting Information

for

Influence of electrospray deposition on C_{60} molecular assemblies

Antoine Hinaut, Sebastian Scherb, Sara Freund, Zhao Liu, Thilo Glatzel and Ernst Meyer

Beilstein J. Nanotechnol. **2021,** *12,* 552–558. doi:10.3762/bjnano.12.45

Additional experimental data

License and Terms: This is a supporting information file under the terms of the Creative Commons Attribution License (https://creativecommons.org/licenses/by/4.0). Please note that the reuse, redistribution and reproduction in particular requires that the author(s) and source are credited and that individual graphics may be subject to special legal provisions.
The license is subject to the *Beilstein Journal of Nanotechnology* terms and conditions: (https://www.beilstein-journals.org/bjnano/terms)

1 Presence of solvent after HV-ESD on Au(111)

It is possible to increase the presence of solvent molecules deposited on the surface during HV-ESD process. Intentionally or not, through high flux or low voltage, large droplets can be created and directed to the surface leading to the deposition of a high amount of solvent molecules. The topography and corresponding excitation images in Figure S1show such a situation where islands of C_{60}, the Au(111) surface, and solvent-covered surface can be identified. The voltage was intentionally modified during the deposition to obtain large droplets.

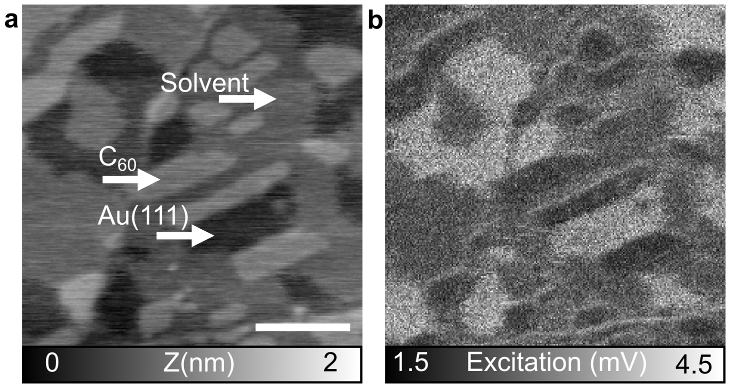

Figure S1: C_{60} on Au(111) surface HV-ESD. (a) Topography and (b) corresponding excitation. Scale bar: 50 nm.

2 TE and HV-ESD on Ag(111)

Figure S2 presents topography ncAFM images acquired on the C_{60}/Ag(111) surface formed after TE (SI Figure S2a) and after HV-ESD (SI Figure S2b). Similar to C_{60} on Au(111), for TE, the growth mode results in C_{60} islands, mainly observed at the step edges of the metal surface. Islands are growing from the top and from the bottom part of the step edges. On the large-scale area, only one island is observed trapped on the terrace (white arrow). Apart from the islands, the Ag(111) surface remains clean. After HV-ESD, the observed islands are well dispersed on the terraces and most of them are found not anchored at step edges. A high-resolution image of the C_{60} lattice in the islands is visible in the inset. Additionally, polluted areas are visible on the terraces, indicated by white arrows.

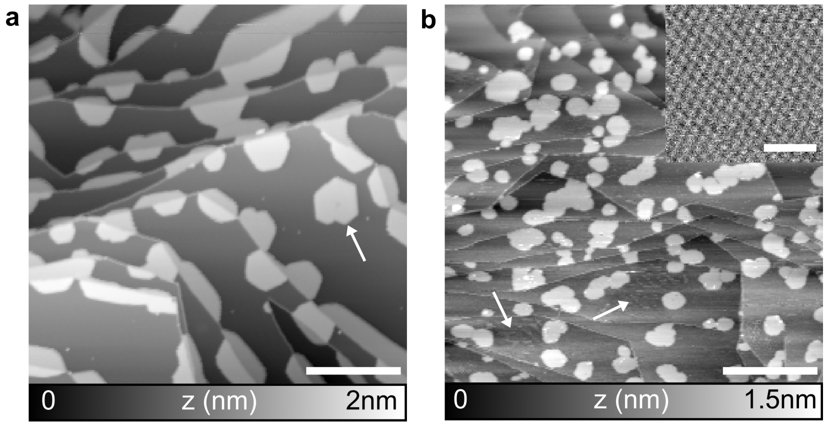

Figure S2: C_{60} on a Ag(111) surface. (a) after TE and (b) after HV-ESD. Scale bar: 400 nm. Inset: High-resolution image of a C_{60} island, scale bar: 5 nm.

3 Defect creation on KBr(001) with HV-ESD

HV-ESD can also lead to the creation of defects on the KBr surface. Similar defects as visible in Figure 4c of the main manuscript can for example be intentionally created and increased in number on the KBr surface. To do so, the high voltage (U) applied between the solution in the emitter and the entrance capillary can be increased. As a result, the energy of the landing species is increased at the impact on the surface. The topography nc-AFM image in Figure S3a was acquired after HV-ESD with $U > 2.5$ kV. A large circular hole, close to 100 nm diameter, is visible on the KBr(001) surface. Surrounding it many rectangular and round islands are observed. The latter, which are displayed with white contrast, are C_{60} islands. The rectangular islands corresponds to KBr material and are better visible in the Figure S3b. The circular crater suggests an impact of a highly energetic droplet. KBr removed from the crater is redistributed closely around the crater.

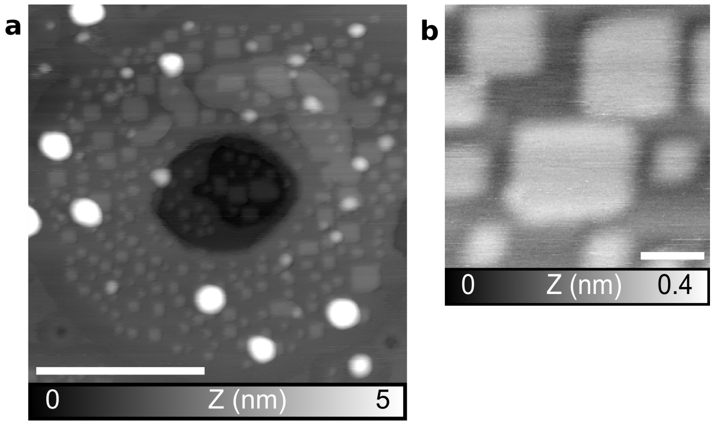

[t]

Figure S3: C_{60} on a KBr(001) surface HV-ESD at high energy. (a) Large topography image of a charateristic crater. (b) Zoom on created KBr islands. Scale bar: (a) 200nm, (b) 10 nm.
